# Positive sentiment and expertise predict the diffusion of archaeological content on social media

**DOI:** 10.1038/s41598-025-85167-z

**Published:** 2025-01-15

**Authors:** Chiara Bonacchi, Marta Krzyzanska, Alberto Acerbi

**Affiliations:** 1 School of History, Classics and Archaeology, University of Edinburgh, Edinburgh, UK; 2 Department of Sociology and Social Research, University of Trento, Trento, Italy

**Keywords:** Archaeology, Psychology and behaviour

## Abstract

This study investigates the dissemination of archaeological information on Twitter/X through the lens of cultural evolution. By analysing 132,230 tweets containing the hashtag #archaeology from 2021 to 2023, we examine how content and context-related factors influence retweeting behaviour. Our findings reveal that tweets with positive sentiment and non-threatening language are more likely to be shared, contrasting with the common negativity bias observed on social media. Additionally, content authored by experts, particularly those with archaeological or historical expertise, is more frequently retweeted than content from popular figures lacking domain-specific expertise. The study also challenges the notion that pseudoarchaeology spreads rapidly and caution against overestimating its impact. Our results align with other studies on the spread of misinformation and “toxic” behaviour on social media, showing that the sharing of negative and hostile content by a vocal minority of users is mediated by other factors pertaining to the context of the communication. These insights underscore the nuanced dynamics of archaeology communication, emphasizing the importance of expert-led and positively charged narratives in engaging the public on social media.

## Introduction

This article examines the factors impacting the sharing of archaeological information on Twitter/X through the theoretical lens of cultural evolution. Since its antiquarian roots, archaeology has been a powerful source of knowledge about the human past. In the 1800s, it was featured in learned lectures, gentlemen’s magazines and the early press^1^. By the mid-1950s, archaeology had become a popular subject on television in the UK and the US, two pioneers of public service broadcasting^2^. Since the 1990s, it has appeared in video games such as Sid Meier’s *Civilisation VI*, *Minecraft* and *Assassin’s Creed Odyssey*, through which millions of people now spend billions of hours interacting with the past^3^. Today, archaeological information also circulates on social media, where users share it with others who may be unknown to them and located anywhere in the world.

Social media is profoundly transforming communication by expanding the network of individuals who can publish, select, modify and disseminate information online^4^. Among the various social media platforms available, Twitter/X has played a key role in facilitating peer-to-peer and public engagement with archaeology and other scientific subjects^5,6^. This platform is widely used by professionals in higher education, museums, galleries, libraries, heritage sites and management bodies. Studying science communication on Twitter/X is also important because the findings can be applied to emerging social media like Threads, Mastodon, and Bluesky. These follow in the footsteps of Twitter/X, by adopting structures and interfaces that facilitate open forms of following users, posting and re-sharing content. For these reasons and due to the greater accessibility of Twitter/X data compared to other platforms, a substantial body of literature has explored how archaeology is communicated on Twitter/X.

Publications on this topic typically have focused on either theoretical discussions or primarily qualitative empirical research. They often evaluate projects and case studies relating to professional practice and networking^7,8^, public education, and outreach^9,10^. Other studies have analysed socially divisive uses of archaeology on Twitter/X^11–14^. In contrast, only a few works assess the features of Twitter/X communications of archaeology quantitatively, using social media data. Marwick^15^ was the first to apply text mining techniques to examine the issues appearing in Twitter debates held during the 109th Annual Meeting of the American Anthropological Association (AAA). Subsequent publications have quantitatively analysed tweets to evaluate education programmes^16,17^ and examine how archaeology and heritage are used to support political positions, especially in populist, nationalist and far-right discourse^6,18,19^. Among these, a study revealed how ‘pseudoarchaeology’ content defending ‘ancient alien origins’ has been leveraged to communicate white supremacist views^20^.

Researchers have also formally analysed sentiment in archaeology-themed micro-blogging, producing different results depending on the communication context. The studies assessing sentiment in people’s responses to outreach activities have found sentiment scores to vary based on the initiatives and their timing. However, these findings are tied to specific public engagement actions and less informative about the sentiment of appealing archaeological information shared on social media. Studies of tweets commenting on heritage sites have shown that sentiment can remain positive even during challenging times, such as the Covid19 pandemic^21^, and that it may change in relation to visitors’ experiences^22^. Conversely, a recent study found that Facebook posts mentioning the ancient past to express political views tended to have mostly negative and extreme sentiment^23^, more so than posts without such mentions. Therefore, it may be hypothesised that X posts referencing archaeology in political debates may also be predominantly negative and extreme.

Finally, we have limited knowledge about the social actors most influential in spreading archaeological information on Twitter/X. Archaeology-themed Twitter/X posts seem more likely to be reposted if authored by users with public roles, including public intellectuals, media personalities, politicians, and activists^24^. However, the impact of expertise versus general prestige (not related to specialist knowledge) remains unclear.

The literature summarised above highlights the importance of studying social media communications of archaeology, especially on Twitter/X. Yet, existing publications have mainly focused on rather narrow communication contexts and have not assessed, through a coherent theoretical framework, how both content-related and context-related features influence the sharing of archaeological information on Twitter/X.

### Theoretical background and predictions

Cultural evolution is a framework developed to understand how culture changes and spreads over time (starting from^25,26^), including in digital and online media^4^. One research thread in cultural evolution seeks to identify two types of factors that make certain cultural traits more successful in spreading than others—both are considered in this study. *Content*-related factors consist in intrinsic features of cultural traits such as the presence of attractive attributes, or their ease to transmit (see e.g.^27^). *Context*-related factors are social forces such as preferentially copying and transmitting traits that are popular (conformity bias) or held by individuals deemed prestigious (prestige bias).

Our analysis is centred particularly on two context-related variables: the general prestige of users (measured through the number of followers) and domain-specific prestige (archaeological expertise identified with a topic model of twitter/X user profile bios). Brand, Mesoudi and Morgan^28^ emphasise the importance of distinguishing between general prestige bias in social learning and domain-specific prestige bias, which occurs when ‘individuals choose to learn from a prestigious model only within the domain of expertise in which the model acquired their prestige’ ^[28, p. e0255346]^. Differentiating between the two is paramount, given that cultural evolution research has hypothesised that expertise might not be favoured in online environments as it becomes more difficult to recognise^29^. Furthermore, the effect of general prestige—as in the case of generic social media “influencers”—is debated in cultural evolution. Some argue that we rarely copy traits from prestigious individuals if those traits are not advantageous, or neutral, to us^4^.

Regarding content features, we consider two characteristics that were shown to favour information sharing and that may be important also for the communication of archaeology on Twitter/X. These are the presence of threatening language—found to affect information spread in cultural transmission experiments (e.g.^30^)—and sentiment (see e.g.,^31–35^. Generally, studies reveal an advantage for negative information, but also that it is dependent on the communication subject/domain^36^ or the behaviour of a minority of popular users^37^. Therefore, it is important to establish the impact of these content features alongside the specific topics covered in social media communications. To achieve this, we use topic modelling to identify themes in tweets/X posts and determine how they influence information sharing.

This cultural evolution framework guided the analysis of 132,230 tweets and quote retweets containing the hashtag ‘#archaeology’, written in English and published between 01.01.2021 and 31.01.2023. Focusing on tweets with hashtag ‘#archaeology’ allowed us to concentrate on posts flagged by users as being about archaeology^38^. Based on the archaeology and cultural evolution literature reviewed, we formulated a series of hypotheses about how content and context-related features affect the number of retweets in our dataset:

1. Twitter/X posts with threatening language are more likely to be shared.
2. Twitter/X posts with negative sentiment are more likely to be shared.
3. Twitter/X posts authored by users deemed prestigious are more likely to be shared.

We could not predict the impact of authors’ expertise on post sharing on Twitter/X, and tested the effect of this factor alongside the three hypotheses using a multilevel Bayesian regression model that included relevant variables (threat, sentiment, number of followers, topic and user category) as predictors.

## Results

### Content-related features

#### Threat

Threat level is measured as the number of threat words in a tweet (see Materials and methods). It has a negative effect on the retweeting of a tweet ${\beta }_{TL}=-0.27$ with an estimated decrease of about 0.07 units in log count of retweets for tweets with one threat word, and of about 0.27 units with the maximum number (5) of threat words present. Since we use the Poisson model with log-link, this decrease in the expected number of retweets is non-linear on the absolute scale.

#### Sentiment

The sentiment of tweets was estimated within a range from −1 to 1 using the Valence Aware Dictionary and sEntiment Reasoner (VADER) (see Materials and methods). The positive coefficient value for sentiment (${\beta }_{S}=0.08)$ indicates that the number of retweets increases with higher sentiment scores. Specifically, the mean expected number of retweets differs by 1.6 units on the log scale between tweets with the highest positive (1) and lowest negative (−1) sentiment scores. This aligns with the negative effect of threat words, suggesting that positive tweets without threatening language are more likely to be shared. Furthermore, given the partial overlap between words associated with threat and those with negative sentiment, the results highlight the positive effect of positive sentiment, even when accounting for the negative impact of threat words on retweeting.

#### Themes

**Fig. 1:**
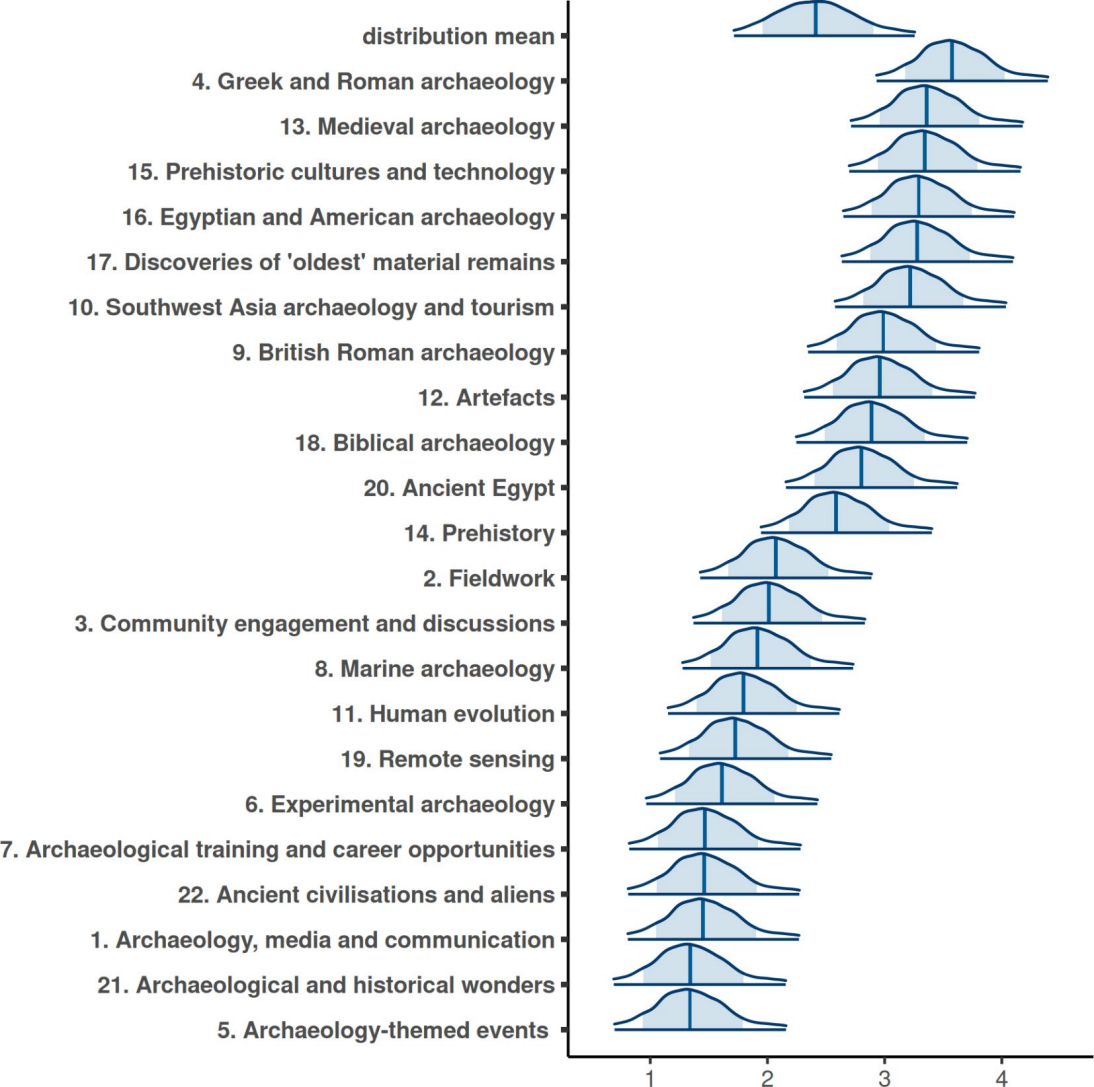
Posterior predictions for the intercept values of different topics in the tweets and estimated mean of their distribution. The blue area indicates the 80% confidence intervals, while the edges of the distribution show the 95% confidence intervals.

The intercepts for different topics, identified via topic modelling (see Materials and methods), reveal how the subject of the tweet affects retweet numbers. The mean coefficients for different topics range from 1.36 to 3.59, with considerable overlap in their confidence intervals (Fig. 1). The overlap indicates uncertainty about the relative popularity of topics with similar coefficient values. However, there is no overlap between the 95% confidence intervals for the top and bottom five topics, and these show the archaeological themes that are most and least likely to be shared (Fig. 1).

The five topics most likely to be retweeted are ‘Greek and Roman archaeology’ (topic 4), ‘Medieval archaeology’ (topic 13), ‘Prehistoric cultures and technology’ (topic 15), ‘Egyptian and American archaeology’ (topic 16), and ‘Discovery of oldest material remains’ (topic 17).

These themes were identified based on the most relevant terms ($\lambda$=0.6) in the topic model (see Materials and methods), encompassing place and period identifiers as well as commonly associated finds. Conversely, the least popular topics were ‘Archaeology-themed events’ (topic 5), ‘Archaeological and historical wonders’ (topic 21), ‘Archaeology, media and communication’ (topic 1), ‘Ancient civilisations and aliens’ (topic 22), ‘Archaeological training and career opportunities’ (topic 7).

### Context-related features

#### Prestige

General prestige—measured through number of followers—has a positive effect on whether a tweet will be retweeted at all, as indicated by the negative coefficient (${\beta }_{p}=-0.77)$ that predicts the probability of zero retweets within the ‘zero-inflated’ part of the mixture model used. Once this effect is accounted for, the number of followers appears to have little (negative) to no effect on the number of retweets (${\beta }_{F}\sim 0.00)$.

#### Expertise

**Fig. 2:**
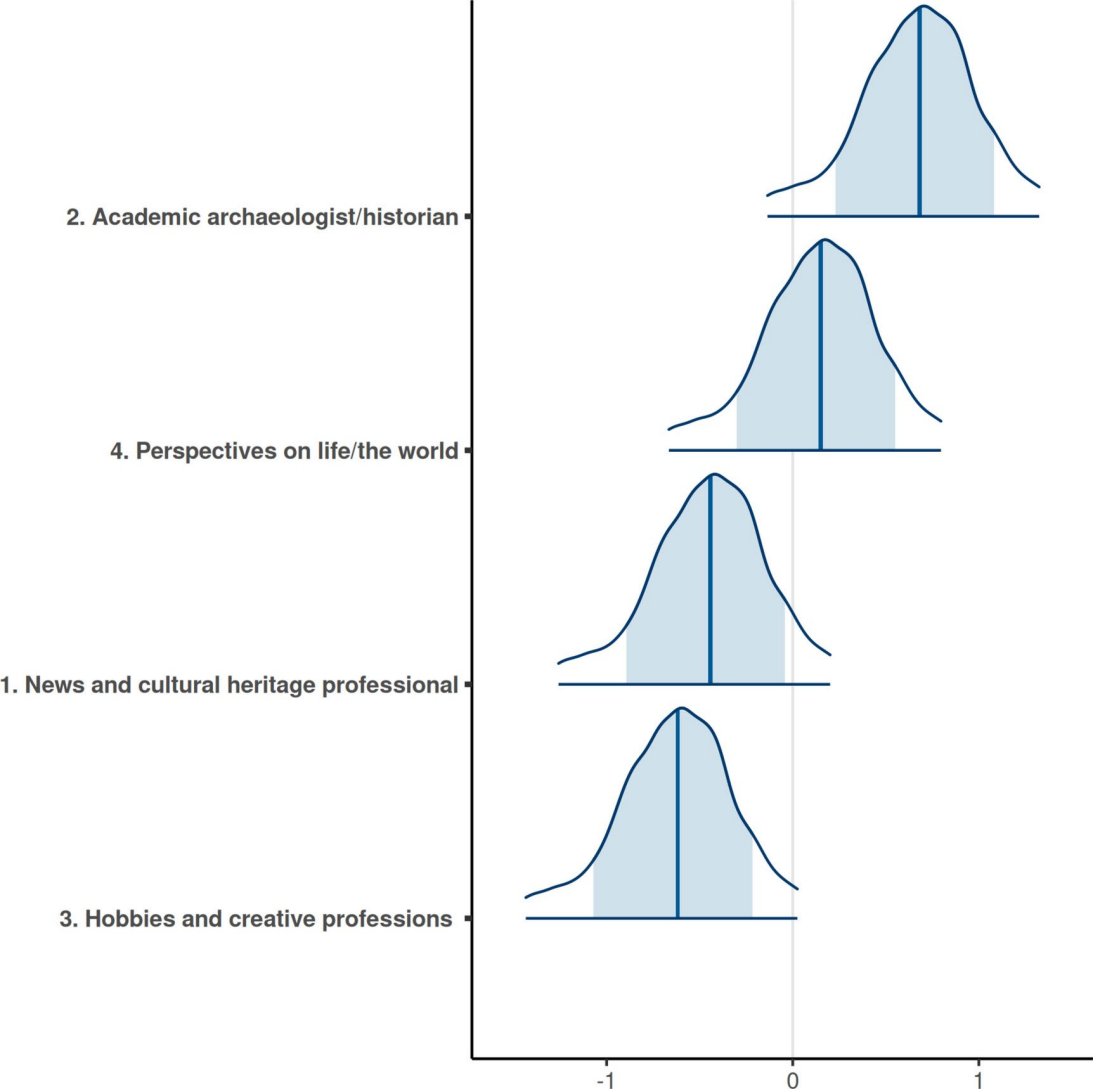
Posterior predictions for the intercept values of different user identities. The blue area covers the 80% confidence intervals, while the distribution boundaries cover 95% confidence intervals. The mean of the distribution from which the intercepts were sampled was set to 0. This is because, in models with two types of varying intercepts, estimating a separate mean for each could lead to an unidentified model.

The intercepts for different user categories, identified via topic modelling (see Materials and methods), reveal differences in retweet numbers based on the authors’ identities. The mean intercept values for user categories range from −0.64 to 0.66. While the confidence intervals for individual intercept predictions overlap significantly (Fig. 2), the mean of the posterior distributions shows that tweets are most likely to be retweeted when authored by users who describe themselves as archaeologists or historians in their profile descriptions. This category is followed by accounts offering specific perspectives on life or the world, news and cultural heritage professionals, and creative professions. This suggests that professionalism—particularly academic expertise related to archaeology and history—has a positive effect on the dissemination of information.

## Discussion

This study shows that Twitter/X posts about archaeology are more likely to be shared when they express positive sentiment and use non-threatening language. This finding is crucial for understanding the role of emotion in cultural evolution online and in public engagement with archaeology. While previous studies have found sentiment to be predominantly negative and extreme in political discourse mentioning the ancient past on social media^23^, our analysis suggests that, beyond discussions of nationalism, populism, and far-right ideologies, positive content about archaeology is more likely to be shared.

In political science and heritage literature, positivity bias is explained through the concepts of nostalgia^39,40^ and retrotopia^41^. These have been defined as responses to ‘modern malaise’^39^, [p. 31] and ‘liquid times’^42^. Sedikides and Wildschut^43^, [p. 48] argue that ‘Nostalgia—the sentimental longing for one’s past—is a self-relevant, albeit deeply social, and an ambivalent, albeit more positive than negative, emotion.’ From this perspective, the past is seen as offering a comforting refuge in a world shaped by neoliberalism and globalisation, two phenomena considered responsible for increasing a general sense of uncertainty^44^. In these circumstances, as Bauman notes, retrotopias represent a controlled longing for the past, and can be more desirable than unpredictable future utopias^41^.

Our findings align with the results of a recent European survey showing that public attitudes towards archaeology are generally positive. However, while people may regard archaeology as valuable^45^, [p. 105], their relations with specific pasts can vary. The social value of heritage is ‘a collective attachment to place that embodies meanings and values that are important to a community or communities’^46^, [p. 22]. Yet, this attachment can be associated with positive or negative emotion. It ‘encompasses the ways in which the historic environment provides a basis for identity, distinctiveness, belonging and social interaction’^46^, [p. 22]. In some instances, those identities are built through feelings of belonging to ‘difficult heritage’ that evokes painful memories (see, for example,^47,48^) for certain individuals. There are several possible examples, ranging from Magdalene laundries^49^ to colonial heritage^50^. It would thus be a mistake to equate the positive sentiment of archaeological information with which users preferentially engage in our study with the social value of archaeology or heritage *tout court*.

Furthermore, as Wetherell et al.^51^, [p. 12] put it:

‘A strong emphasis has traditionally been placed on the sense of belonging engendered by heritage, but heritage is always a simultaneous act of inclusion and exclusion. Exclusion may occur through the simple act of demarking what is or is not someone’s heritage or through active attempts to forget or obscure diversity and difference by the very assertion of monolithic ideas of national or ‘universal’ heritage.’

One might hypothesise that people who engage with positively polarised information about #archaeology, feeling a sense of belonging to an ‘imagined community’, could use that same information in other contexts—such as political discussions on social media—to exclude specific groups through negative and threatening language. Although negativity bias does not seem to affect those connecting with archaeological content via #archaeology, participants in these interactions may still preferentially share negatively polarised posts in political debates where archaeology serves more as a rhetorical tool.

The fact that positive sentiment predicts engagement with archaeology-themed tweets may seem at odds with the general notion of a negativity bias in social media information diffusion^32–35^. However, our finding helps to contextualise the extent of this negativity bias. While a psychological tendency to pay preferential attention to negative information is uncontroversial, its effects on the spread of information are mediated by many variables. A positivity bias in *sharing* information, especially non-anonymously and within networks of known people, can counterbalance a negativity bias in *attending* to information, depending on the situations and the affordances of specific social media (see e.g.^52^). For example, if most information is negative, positive information becomes more surprising and, thus, catchy^36^. Furthermore, niche communities centred on specific topics, as in our case, can maintain a positive attitude within them. Future studies on the circulation of information on social media should consider how negativity bias interacts with all these different factors.

As regards favoured themes, content highlighting the ‘oldest’ age of discoveries (topic 17) is among the most retweeted. This result is consistent with the idea that remote pasts are valued because they feel out of reach and, thus, safe. The appeal of superlatives emphasising the ancient character of finds was hypothesised and discussed anecdotally for other forms of archaeological communication, such as the press^53^, but had never been tested before. Comparing the five most retweeted topics with the five least retweeted ones also shows that content appealing to narrower, more specialised or interested audiences is less likely to be reposted. This includes tweets about training and career opportunities, media and communication, and archaeology-themed events.

An additional and unexpected finding is that content focused on pseudoarchaeology is among the least likely to be shared. Twitter/X posts about ancient civilisations and aliens (topic 22, with most salient terms comprising UFO, mar, aliens, NASA, ESA, ancient aliens, etc.) are reposted less than information on prehistory, Greek and Roman archaeology, Egyptian and American archaeology, and the Middle Ages. Since the dataset includes only pseudoarchaeology tweets that use the hashtag #archaeology, it may not fully represent how likely such content is to be shared in general. However, this result suggests the need to tone down concerns about the rapid and preferential spreading of pseudoarchaeological claims. It indicates that, relative to mainstream information, pseudoarchaeology remains quite niche and siloed, at least within the Twitter community using the archaeology hashtag.

Misinformation does not spread successfully online as often as sometimes feared^54^. When it does spread effectively, it is because it can be crafted to include cultural traits that usually favour the dissemination of information, such as negative emotion and threatening language^31^. However, these traits are not favoured in all communication contexts, as demonstrated here.

Finally, we discovered that, contrary to what has sometimes been hypothesised, content posted by experts in archaeology or history—especially academics—is more likely to be reposted. In contrast, general prestige, measured in terms of follower count on Twitter/X, influences whether a tweet is shared at all but, beyond that, has negligible effect on information spread. This suggests that the idea that expertise is necessarily difficult to recognise on social media due to ‘aggregate patterns of high popularity bias and low transparency of information’^29, [p. 189]^ may be unsupported. Our results align with experimental work^28, [p. 1]^ which indicates that people’s choice between domain-specific and domain-general prestige bias depends ‘on their experience and understanding of the relationships between domains’. In social media environments too, in sum, we can recognise and pay attention to domain-specific experts over generically prestigious individuals^4^.

## Conclusion

This study sheds new light on the reasons behind the appeal and spread of archaeological content on social media. Mainstream archaeology, authored by experts, is more likely to be shared on Twitter/X than pseudoarchaeology. Furthermore, content with positive, non-threatening language is favoured. This shows that negative emotions and misinformation do not have an inherent advantage in social media diffusion. The success of archaeological information depends on the broader subject or the target community, and the variation in the effects of these offers a promising avenue for further research. Our findings are in step with other studies on the spread of misinformation on social media^55^ or “toxic” behaviour^56^, showing that the sharing of negative and hostile content by a vocal minority of users is mediated by other factors pertaining to the context of the communication. These divisive voices, however, can disproportionately affect the experience of social media for most people.

## Materials and methods

### Identifying context-related and content-related features

From 01.02.2023 to 08.02.2023, we used the R library *academictwitteR* to collect historical data from Twitter/X via the Academic API available at the time^57^. This API provided access to the entire Twitter archive, allowing us to extract tweets containing the search term ‘#archaeology’ within a defined time interval. The initial query returned all public Twitter/X posts that contained the specified keyword, but our analysis focussed on 132,230 original tweets and quote retweets (retweets with comments), excluding replies and retweets. Focusing on ‘#archaeology’ enabled us to specifically target posts flagged by users as relevant to archaeology. We also extracted metadata for each tweet author, including one of the context-related predictors used in the model: the *follower count*. Other context-related and content-related features were defined or quantified using natural language processing techniques.

Topic modelling was applied to user profile descriptions to define *user identities* and to the text of tweets to identify the themes featured. The models were constructed with Latent Dirichlet Allocation (LDA)—a popular algorithm for automated topic discovery in large collections of unstructured text^58^. We implemented this model using Python’s *gensim* library^59^ and used the *pyLDAvis* library^60^ to generate interactive visualisations that aid in interpreting topics. In LDA, each text in a corpus (here, each tweet) is a mix of various topics, represented by collections of terms present in the corpus and expressed as term probabilities. The algorithm assumes that, to generate each document, topics are first selected from a random probability distribution within the corpus. Then, terms are generated based on their probability distribution within the selected topic. The model reverse-engineers this process to estimate topic probabilities for each document and term probabilities for each topic^58^.

In LDA, the number of topics needs to be pre-defined. The optimal number of topics *n* was determined by constructing models with numbers of topics between 2 and 29, and selecting the ones with the best coherence scores (*Cv*)^61^. *Cv* reflects semantic similarity between the highest probability terms in each topic, ranging from 0 to 1. Higher scores indicate greater similarity, facilitating topic interpretation. The best model for user descriptions had four topics, while tweet texts had 22 topics. Topics were ordered according to how prevalent they were in the texts, with topic 1 appearing the most. Authors independently labelled topics for user identities and tweets based on the most relevant terms for each topic. Term relevance was calculated as the ratio between the weighted average of the log probability of the term under the topic and the log ratio of the term’s probability under the topic to its marginal probability in the entire corpus, with the weight ($\lambda$) set to 0.6 as recommended by Sievert and Shirley^60^. We then discussed the independently assigned labels and reached a consensus on the most accurate ones. For example, the 20 most relevant terms for topic 12 were: ‘pottery’, ‘content’, ‘reddit’, ‘artifactporn’, ‘content_reddit’, ‘ceramic’, 'india’, ‘archaeology’, ‘plate’, ‘clay’, ‘algeria’, ‘pot’, ‘amp’, ‘sherd’, ‘century’, ‘pillar’, ‘bottle’, ‘vessel’, ‘mound’ and ‘gobeklitepe’. Most of these terms focus on artefacts—with ‘artifactporn’ being the third most relevant term—and, thus, the topic was labelled ‘Archaeological artefacts’. For each tweet, the tweet and user identity topics with the highest probability were considered dominant.

The *sentiment* of tweets was calculated using the R implementation of Valence Aware Dictionary and sEntiment Reasoner (VADER)^62^, which is designed for microblogging content. VADER combines a pre-defined lexicon of words and their associated valence scores indicating sentiment polarity and intensity with specialised linguistic rules and heuristics that handle features such as capitalisation, punctuation, degree modifiers and emoticons to estimate the sentiment of a text in a range between -1 and 1^62^. We also estimated the *threat level* of each tweet using a threat dictionary to identify the number of terms expressing threatening language^63^.

### Modelling the spread of information

Two context-related features (follower count (*F*) and user identity (U)) and three content-related features (dominant topic (T), sentiment (S) and threat level (TL) of the tweets) were used as predictors for the expected number of retweets (R) in a Zero-Inflated Poisson (ZIP) model within a multilevel Bayesian framework. This model assumes that there are two processes that affect whether a tweet is retweeted: whether anyone saw a tweet, and whether someone who saw it decides to retweet it. Some tweets with 0 retweets may result from the first process (unseen tweets), while others may have lacked appeal. The ZIP model distinguishes these two processes by defining the expected number of retweets (R) as a mixture of the probability that a tweet was unseen ($$p)$$ and the likelihood of the number of tweets defined by the Poisson distribution parameter $$\lambda$$:

$$R_{i} \sim ZIPoisson( p_{i} , \lambda_{i} )$$

The only predictor likely to influence ${p}_{i}$ is the follower count (F), as other variables pertain to the features of the tweets that are relevant only once a tweet has been seen. Therefore, within the model, F is included in the linear equation that defines the log odds of $p$ via the logit link function:

$$logit({p}_{i}) = {\alpha }_{p} + {\beta }_{p}*{F}_{i}$$

The logit link function ensures that $p$, which represents probability, is constrained between 0 and 1. When translated from log odds to raw probabilities, the relationship of *p* with its predictors follows a logistic curve. This part of the model accounts for the excess of tweets with 0 retweets in the dataset, which are unrelated to predictors other than follower count and could otherwise mislead inferences about those predictors.

The expected count of retweets, if the tweet was seen, is defined by the Poisson process. In a Poisson regression, the predictors have a linear relationship with the logarithm of the $\lambda$ parameter (which represents the mean and standard deviation of the Poisson distribution). Hence, the relationship with the number of followers, as well as all the other features of the tweets, is modelled via the log link function:

$$\log \left( {\lambda_{i} } \right) = \tilde{\alpha } + \alpha [T_{i} ] + \gamma \left[ U \right]_{i} + \beta_{F} *F_{i} + \beta_{s} *S_{i} + \beta_{TL} * \mathop \sum \limits_{j = 0}^{{TL_{i} - 1}} \delta_{j}$$

where:

$T$ is the topic.

$U$ is the user identity.

$F$ is the follower count.

$$S$$ is sentiment.

$TL$ is threat level.

$\alpha$ and $\gamma$ represent vectors of intercepts for topics and users,

${\beta }_{F}$ , ${\beta }_{s}$ and ${\beta }_{TL}$ are the parameters that define the relationship between the outcomes and corresponding variables.

Therefore, all the variables within the model have a linear relationship with the number of retweets on the log scale. The priors for the model parameters were as follows:

$$\tilde{\alpha } \sim Normal\left( {3,1} \right)$$

$$\alpha \sim Normal\left( {\tilde{\alpha }, \sigma_{T} } \right)$$

$$\alpha \sim Normal\left( {\tilde{\alpha }, \sigma_{T} } \right)$$

$$\gamma \sim Normal(0, {\sigma }_{U})$$

$${\beta }_{F}, {\beta }_{S} , {\beta }_{T} \sim Normal (\text{0,0.2})$$

$${\beta }_{p} \sim Normal (\text{1,0.5})$$

$${\alpha }_{p} \sim Normal (-\text{1.5,1})$$

$${\sigma }_{T} , {\sigma }_{U} \sim Cauchy(0.0.5)$$

$$\delta \sim Dirichlet\left( {\left[ {2, 2, 2, 2} \right]} \right)$$

In the model, tweet intercepts for tweet and user topics are assumed to originate from the populations of intercepts with a mean and standard deviation estimated by the model. This approach allows for pooling information about intercept values between different tweet topics and user identities, constituting the multilevel component of the model.

The follower count and sentiment scores were standardised prior to the analysis, while the threat level was treated as an ordinal variable. Model fitting was performed with the Markov’s Chain Monte Carlo (MCMC) algorithm implemented in Stan^64^, using four chains with a total of 4000 iterations each, including 1000 warm-up samples. Stan was run through the R interface^64^, and the convergence of Markov’s chains was verified by examining trace plots and the Gelman Rubin diagnostic (Rhat), which should approach 1 when the chains have converged. The data used in the analysis (number of retweets and predictor variables), as well as the code documenting data extraction, management and analysis are provided on Github (see^65^).

## Data Availability

Data can be requested from the corresponding author.
